# Autophagy Inhibitor Chloroquine Enhanced the Cell Death Inducing Effect of the Flavonoid Luteolin in Metastatic Squamous Cell Carcinoma Cells

**DOI:** 10.1371/journal.pone.0048264

**Published:** 2012-10-26

**Authors:** Lien Verschooten, Kathleen Barrette, Sofie Van Kelst, Noemí Rubio Romero, Charlotte Proby, Rita De Vos, Patrizia Agostinis, Marjan Garmyn

**Affiliations:** 1 Dermatology, University Hospitals Leuven, & Department of Oncology, KU Leuven, Leuven, Belgium; 2 Cellular and Molecular Medicine, Laboratory for Cell Death and Therapy, KU Leuven, Leuven, Belgium; 3 Cancer Research UK Cancer Centre Dundee, College of Medicine, Dentistry and Nursing, University of Dundee, Dundee, Scotland; 4 Imaging and Pathology, Translational Cell and Tissue Research, KU Leuven, Leuven, Belgium; University of Colorado, United States of America

## Abstract

**Background:**

Flavonoids are widely proposed as very interesting compounds with possible chemopreventive and therapeutic capacities.

**Methods & Results:**

In this study, we showed that *in vitro* treatment with the flavonoid Luteolin induced caspase-dependent cell death in a model of human cutaneous squamous cell carcinoma (SCC) derived cells, representing a matched pair of primary tumor and its metastasis. Notably, no cytotoxic effects were observed in normal human keratinocytes when treated with similar doses of Luteolin. Luteolin-induced apoptosis was accompanied by inhibition of AKT signaling, and sensitivity decreased with tumor progression, as the primary MET1 SCC cells were considerably more sensitive to Luteolin than the isogenic metastatic MET4 cells. Extensive intracellular vacuolization was observed in Luteolin-treated MET4 cells, which were characterized as acidic lysosomal vacuoles, suggesting the involvement of autophagy. Transmission electron microscopy, mRFP-GFP-LC3 assay and p62 protein degradation, confirmed that Luteolin stimulated the autophagic process in the metastatic MET4 cells. Blocking autophagy using chloroquine magnified Luteolin-induced apoptosis in the metastatic SCC cells.

**Conclusion:**

Together, these results suggest that Luteolin has the capacity to induce selectively apoptotic cell death both in primary cutaneous SCC cells and in metastatic SCC cells in combination with chloroquine, an inhibitor of autophagosomal degradation. Hence, Luteolin might be a promising agent for the treatment of cutaneous SCC.

## Introduction

Recent biochemical en preclinical studies provide evidence that flavonoids, bioactive compounds which can be derived from a variety of plants, possess multiple pharmacological activities, including antioxidant, anti-inflammatory and anticancer effects. Luteolin (LUT), one of the most common flavonoids, has the ability to induce apoptosis, to prevent carcinogenesis and to reduce tumorigenesis, which suggests its potential use as a therapeutic treatment [1], even in multidrug resistant cells [2].

Next to their role as conventional hydrogen-donation antioxidants [3], [4], growing data have revealed that flavonoids exert their effects predominately through modulation of protein kinase signaling pathways [5], [6]. LUT, amongst other flavonoids, acts as a competitive inhibitor of protein kinases (such as AKT, MEK1, PKC) [7], probably by direct binding to their ATP binding site, thereby altering the phosphorylation status and influencing multiple cell signaling pathways [5]. Since the inhibition of protein kinases appears to be an important strategy for cancer chemoprevention and cancer therapy [8], flavonoids have emerged as interesting biomolecules in that field [6].

Noteworthy, the activities of flavonoids appear to be very cell type dependent. Indeed, we recently discovered that LUT increased the resistance of normal human keratinocytes (NHK) to ultraviolet (UV) B-irradiation, a potent risk factor for skin carcinogenesis. However, LUT has no photoprotective effect on UVB-induced cell death of malignant keratinocytes derived from human cutaneous squamous cell carcinoma (SCC) [9].

SCC of the skin is a common cancer within the Caucasian population. The incidence of SCC is increasing worldwide, with epidemic proportions in Australia. Early primary SCC of the skin has a high curability and relatively low overall metastatic rate of 3 to 5%. However, certain tumor and patient characteristics (such as the use of immunosuppressive medication [10]) predispose patients to the development of nodal disease and distant metastasis, which portends a poor prognosis with 5 year survival ranging from 14 to 39% regardless of the treatment used [11].

AKT (also known as protein kinase B (PKB)) is a known molecular target for cancer drug development, since aberrations in AKT signaling are frequently observed in human malignancies, including in SCC of the skin [12], [13]. In dermatologic oncology, the PI3K/AKT pathway is known to have a role in both the development of skin cancer as in the generation of resistance towards therapeutic drugs [14], [15]. AKT is a serine/threonine protein kinase and a central node in cellular signaling; crucial in survival, proliferation, metabolism and migration. Deregulation of PI3K signaling and constitutive AKT expression is reported in many cancers, making it an ideal therapeutic target [12]. At the cellular level AKT is recruited to the plasma membrane upon stimulation with growth factors, cytokines and other factors. Phosphorylated (Ser473 and Thr308) and activated AKT phosphorylates a multitude of substrates, including the pro-apoptotic protein Bad and the mammalian target of rapamycin (mTOR) [16], considered as the master regulator of macroautophagy (hereafter called autophagy).

While apoptosis is globally known as an active, programmed and very regulated form of cell death executed by caspases [17], autophagy is highly considered as a survival process, but can be involved in mediating non-apoptotic cell death under certain circumstances [18], [19]. Furthermore, the regulation of autophagy and apoptosis is intimately connected; autophagy can inhibit apoptosis [20], but can also lead to apoptotic cell death [21]. The process of autophagy starts by the formation of double membrane vacuoles, called autophagosomes, capturing long-lived, misfolded or damaged proteins and aberrant organelles. Those cargo-containing vesicles fuse with lysosomes, leading to the degradation and recycling of cellular constituents [22].

In our laboratory, we use a unique model of isogenetic cutaneous SCC cell lines; MET1 and MET4 cells are derived from respectively a primary invasive cutaneous SCC and its lymph node metastasis from an immunosuppressed patient [23]. Using this model system, we reported previously AKT activation parallels skin tumor progression and increased resistance towards cisplatin, a chemotherapeutic agent currently used in the treatment of SCC [24]. Specific inhibition of AKT sensitized the primary tumor cells (MET1) to cisplatin. The metastatic MET4 cells, on the other hand, showed reduced sensitivity together with the induction of autophagy.

In this study we investigated the potential therapeutic effect of LUT on MET1 and MET4 cells representative for different stages of SCC carcinogenesis. Even though we demonstrated that the flavonoid LUT is a strong apoptosis inducing agent in primary SCC cells (MET1), MET4 cells showed higher resistance to LUT due to the induction of autophagy as survival mechanism.

## Materials and Methods

### Reagents and antibodies

Luteolin (Sigma, St. Louis, MO) was dissolved in DMSO and kept protected from light. The concentrations LUT used in the experiments varied between 10–100 µM. In case not specified in the figure legend, values refer to concentration in µM. We purchased the Parp and p62 antibody from BD Biosciences, anti-LAMP-2 antibody from Abcam (Cambridge, UK) and the anti-LC3 antibody from NanoTools (Teningen, Germany). Antibodies against the cleaved form of caspase 3, against phospho-AKT (Ser473), AKT, phospho-mTOR (Ser2448), phospho-p70S6K (Thr389), p70S6K and the secondary HRP-labeled goat-anti-mouse and goat-anti-rabbit antibodies, were obtained from Cell Signaling Technology (Beverly, MA). The primary antibody against actin (JLA20) and against LAMP-1 (H4A3) were purchased from Developmental Studies Hybridoma Bank at the University of Iowa. 3-(4,5-Dimethylthiazol-2-yl)-2,5-diphenyltetrazolium bromide (MTT), Acridine Orange (AO), 3-methyladenine (3-MA) was from Sigma-Aldrich (Saint Louis, MO). zVAD-fmk was from Bachem (St. Helen's, UK) and AKT inhibitor VIII was from Calbiochem (San Diego, CA). The secondary AlexaGreen488-labeled anti-mouse antibody and Prolong Gold Antifade Reagent were purchased from Molecular Probes (Invitrogen-Life technologies) transfection reagent was purchased from Roche (Germany).

### Cell culture

MET1 and MET4 cell lines were respectively derived from a primary cutaneous invasive SCC and from its metastasis within left axillary lymph nodes from the same patient [23]. The A253 cells were derived from a head and neck [25]. Primary human keratinocytes were isolated and pooled from foreskins of different donors (less than 5 years) as described [26]. All cells were grown as previously described [9] in a 37°C incubator at 5% CO_2_.

Expression of constitutively active AKT was obtained by transfection of MET1 and MET4 cells with pLNCX-HA-myrAkt (MyrAKT-HA) using Fugene HD (2/5 ratio) as per the manufacturer's instructions (Roche, Germany). Transfected cells were pooled and reseeded 24 hours after transfection in order to start the experiment with a homogeneous pool of successfully transfected cells.

### Viability assays

Metabolic activity was assessed using 3-(4,5-Dimethylthiazol-2-yl)-2,5-diphenyltetrazolium bromide (MTT) as described earlier [9]. In addition, cell viability was assessed using the trypan blue exclusion assay. Cells were seeded in plates and treated as described. Cells were harvested using trypsin-EDTA (Invitrogen, Merelbeke, Belgium), resuspended in PBS and analyzed using a Countess Cell Counter (Invitrogen, Merelbeke, Belgium).

### Western blot analysis

After the indicated treatments, cell lysates were prepared, separated and analyzed as previously reported [9]. In brief, after determination of the protein concentration using the BCA Protein Assay Reagent (Pierce Chemical Company, Rockford, IL, USA), equal amounts of protein (20–50 µg) from each sample were separated by electrophoresis through SDS-PAGE gels (4–12% Tris-HCl, Nupage, Invitrogen, Merelbeke, Belgium) and transferred to Hybond-C Super membrane (Amersham Pharmacia Biotech, Piscataway, NJ). Protein bands were visualized using enhanced chemiluminescence as described by the supplier (Amersham Pharmacia Biotech, Piscataway, NJ).

### Measurement of caspase activity

Cells were treated as indicated, harvested via trypsinisation and counted using Countess cell counter (Invitrogen). Equal amount of cells (50.000 per well) were transferred to a white 96 well plate. Caspase activity was detected using the Caspase 9 glo assay (Promega, Madison, WI) according to manufacturer's protocol. Readout was done using a VICTOR^3^ Multilabel counter (Perkin Elmer, Waltham, MA) and Wallac software 60 minutes after addition of the substrate.

### Cell death assays

Detection of DNA fragmentation (SubG1 cells) was performed in propidium iodide stained cells as previously described [9].

*In vitro* determination of cytoplasmic histone-associated-DNA-fragments (mono- and oligonucleosomes) was performed using Cell Death Detection ELISAPLUS (Roche Applied Science, Mississauga, ON, Canada). Both adherent and floating cells were collected and the ELISA was then carried out as per the instructions of the manufacturer.

### Fluorescent detection of lysosomes and autophagy

To detect and quantify AVOs, cells were stained with acridine orange (final concentration of 1 μg/ml in PBS, 15 min). Cells were then analyzed with a fluorescent microscope (Olympus, Cell software) or trypsinized and analyzed by flow cytometry (FACScan, Becton Dickinson; CellQuest software).

To visualize lysosomes, cells were fixed with paraformaldehyde (4%; 20 min), permeabilized with ice cold methanol, blocked with 10% FBS and 1% BSA in PBS and subsequently incubated overnight with primary anti-LAMP-1 or anti-LAMP-2 antibody. After incubation with the AlexaFluor488-labeled secondary antibody, cells were post-fixed with paraformaldehyde, counterstained with DAPI, mounted with Prolong Gold Antifade reagent and analyzed using an Olympus Fluoview FV1000 confocal microscope (Melville, NY).

To analyze autophagic flux, MET4 cells were transfected with mRFP-GFP-LC3 (also known as tandem fluorescence-LC3, tf-LC3) expressing plasmid and Fugene HD (2/6 ratio) as per the manufacturer's instructions (Roche, Germany). Cells were pooled, seeded in chamber slides and left untreated (to avoid transfection-induced autophagy). Autophagic flux was determined by evaluating the punctuated pattern of GFP and mRFP (punctae/cell were counted). Analysis of fluorescence was done on an Olympus (Aartselaar, Belgium) cell imaging station using Cell M software.

### Statistical analysis

The data were expressed as means ± S.D. Statistical analysis was performed by using Student's t-test (two-tailed). The criterion for statistical significance was taken as (p<0.05) unless stated otherwise.

## Results

### Luteolin induces apoptotic cell death in SCC cells, but sensitivity decreases with SCC tumor progression

**Figure 1:**
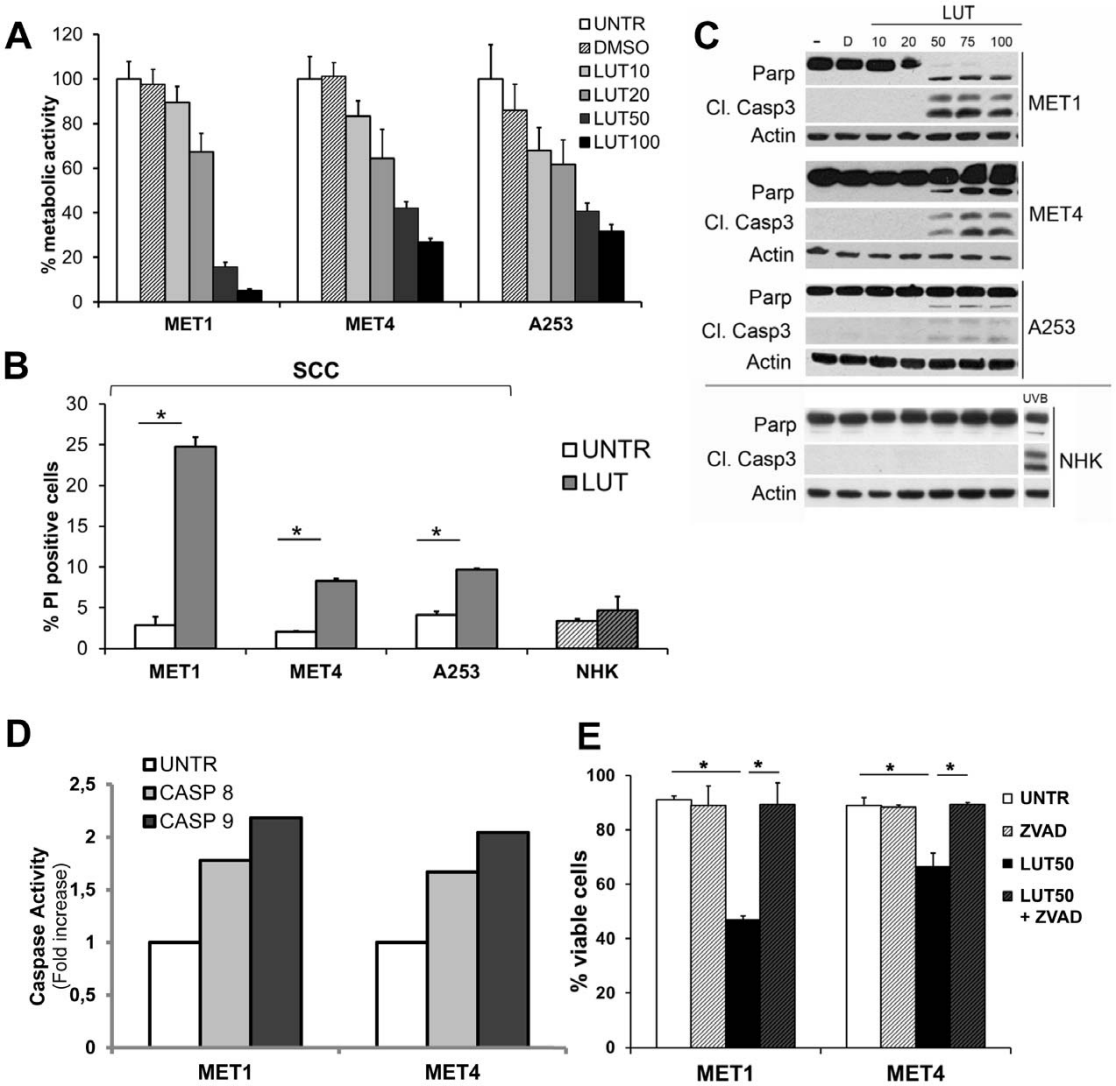
LUT induces apoptotic cell death specifically in malignant keratinocytes. **A.** Detection of metabolic activity by MTT assay, 24 hours after treatment with LUT (10–100 µM) or DMSO (equal amount as highest LUT concentration) in different SCC cell lines. (Representive experiment out of 3, n = 16) **B.** Flow cytometric analysis of propidium iodide (PI) stained cells, 24 hours after treatment with LUT (50 µM). PI-positive cells = dead cells. (Representive experiment is shown, n = 3) **C.** Detection of caspase signaling by western blot analysis 24 hours after different concentrations of LUT (10–100 µM). UVB (120 mJ/cm^2^) was used as a positive control for cell death in NHK. **D.** Luminescent detection of caspase 8 and caspase 9 activity 24 hours after treatment with 50 µM LUT. Fold increase values against untreated control are shown. (Experiment performed three times in duplicate) **E.** Measurement of viability by trypan blue exclusion assay, 24 hours after treatment with LUT (50 µM) and zVAD-fmk (50 µM).

Recent studies indicate that LUT has potential anticancer effects in different tumor cell types, however these effects have not been investigated in human cutaneous SCC cells [27]. To address the therapeutic effects of LUT in SCC, we first investigated the cell death inducing effects of LUT on cell lines derived from cutaneous SCC: MET1, MET4 cells and A253 cells. Treatment of those cell lines with LUT (10–100 µM) decreased metabolic activity in all cell lines, but was more pronounced in the primary tumor cells (MET1) compared to the metastatic MET4 in A253 cells *(* *Figure* *1A* *)*. In a concentration range above 50 µM LUT was clearly cytotoxic in all tested SCC cell lines because the percentage propidium iodide (PI) positive cells increased significantly *(* *Figure* *1B* *)*. Interestingly, normal human keratinocytes (NHKs) were completely resistant to the doses of LUT killing the malignant keratinocytes *(* *Figure* *1B*–*C* *)*, in agreement with our previous results on the differential protective effect of LUT against UVB-induced apoptosis in normal and not in malignant keratinocytes [9].

Caspase 3 activation and poly(adenosine diphosphate-ribose)polymerase (Parp) cleavage, two hallmarks of apoptotic cell death, were observed in all SCC cell lines treated with LUT (50 µM or higher), indicating that the decrease in viability upon LUT treatment was due to apoptosis *(* *Figure* *1C* *)*. Cleavage and activation of executioner caspase 3 can be the result of two converging pathways activating specific initiator caspases via the formation of a molecular platform. We used a luminescent assay to measure the activity of caspase 8 and caspase 9, the initiator caspase of the extrinsic and the intrinsic apoptotic pathway respectively. Although the increase in caspase activity after LUT treatment differed between the used SCC cell lines and was LUT dose-dependent, we detected a significant 2- to 3-fold induction of both caspase 8 and caspase 9 *(* *Figure* *1D* *)*, suggesting the involvement of intrinsic as well as extrinsic apoptotic signaling.

Furthermore, zVAD-fmk, a broad spectrum caspase inhibitor, blocked LUT-induced cell death as detected with trypan blue exclusion assay, indicating cell death was caspase dependent *(* *Figure* *1E* *)*. Hence, LUT causes a caspase dependent form of cell death specifically in malignant, SCC-derived keratinocytes.

### LUT mediated apoptosis involves interference with AKT signaling

**Figure 2:**
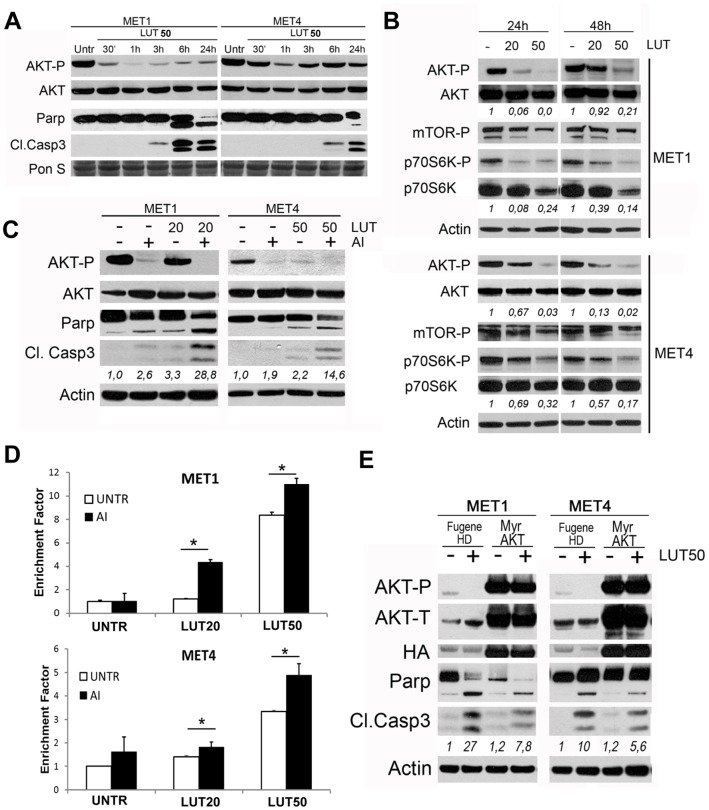
LUT induced apoptosis involves modulation of AKT signaling. **A.** Time dependent effect of LUT on AKT phosphorylation (AKT-P; Ser473) and on apoptosis markers (Parp-cleavage and caspase 3 activation) was studied using western blot in MET1 and MET4 cells **B.** Protein and phosphorylation levels of AKT and its downstream targets in MET1 and MET4 cells treated with LUT (20 and 50 µM) for 24 hours and 48 h obtained using western blot analysis. Numbers, obtained by densitometric analysis of the western blot, indicate the ratio of phosphorylated to total AKT and p70S6 protein. **C.** MET1 and MET4 cells were pre-treated or not with AI (10 µM) for 1 hour and subsequently treated with LUT as indicated. Densitometric analysis of cleaved caspase 3 relative to actin level is shown. A representative blot of at least 3 independent experiments is shown. **D.** Cells were treated with AI and LUT for 24 hours as indicated and the amount of apoptotic DNA was determined by the Cell death detection ELISA as described in Materials and Methods. Experiment was performed twice in duplicate **E.** MET1 and MET4 cells transiently transfected with a construct expressing Myr-AKT-HA, were treated or not with LUT (50 µM) for 24 hours. Fugene HD  = transfectant only; Myr-AKT =  constitutively active AKT). Densitometric analysis of cleaved caspase 3 relative to actin level is shown. A representative blot of at least 3 independent experiments is shown.

Since our previous study revealed that enhanced AKT activation parallels progressive tumor stage and increased resistance of MET1 and MET4 cells to therapeutic drugs [24], we investigated the impact of LUT treatment on the phosphorylation and thus activation of AKT (Ser473) using western blot analysis *(* *Figure 2A* *)*. As soon as 30 minutes after LUT addition, AKT became progressively dephosphorylated in both MET1 and MET4 cells. Interestingly, the exhaustive dephosphorylation of AKT after 6 h LUT treatment in MET1 cells corresponded well with the appearance of apoptotic markers, which suggests the importance of AKT as an inhibitor of apoptosis. To rule out cell specific effect, we treated A431 and A253 cells with increasing amounts of LUT and detected also in these SCC cell lines a progressive down regulation of AKT signaling *(data not shown)*.

To confirm that LUT decreased AKT-mediated signaling, we monitored phosphorylation of the AKT substrate mTOR, and its downstream target p70S6 kinase. LUT clearly inhibited AKT signaling as indicated by the reduced phosphorylation of mTOR and p70S6 kinase in both MET1 and MET4 cells as depicted in *Figure 2B*.

To further investigate whether MET1 and MET4 cells were dependent on AKT signaling for survival, we used a combination of an isozyme-specific AKT1/2 inhibitor (AI) to block basal AKT phosphorylation and LUT. This treatment resulted in additional cell death in MET1 and MET4 cells compared to LUT alone *(* *Figure 2C–D* *)*. In agreement with the fact that MET4 cells are more resistant, a higher LUT dose (50 µM) had to be used to reveal the additive effect on protein level *(* *Figure* *2D* *)*. In addition, we transiently transfected MET1 and MET4 cells with a construct expressing AKT in a membrane-targeted form (myrAKT-HA). The capability of LUT to induce apoptosis active AKT was reduced in both the MET1 and MET4-cells overexpressing constitutively active AKT *(* *Figure* *2E* *)*.

Altogether these data showed that LUT suppressed AKT-signaling in both the primary and metastatic SCC cells. However, MET1 primary tumor cells were more sensitive to LUT-induced apoptosis (*Figure* *1* *&* *2* *)*, suggesting that induction of survival mechanisms in advanced MET4 cells overcome LUT-induced cell death.

### LUT treatment increased the lysosomal compartment in MET4 cells

**Figure 3:**
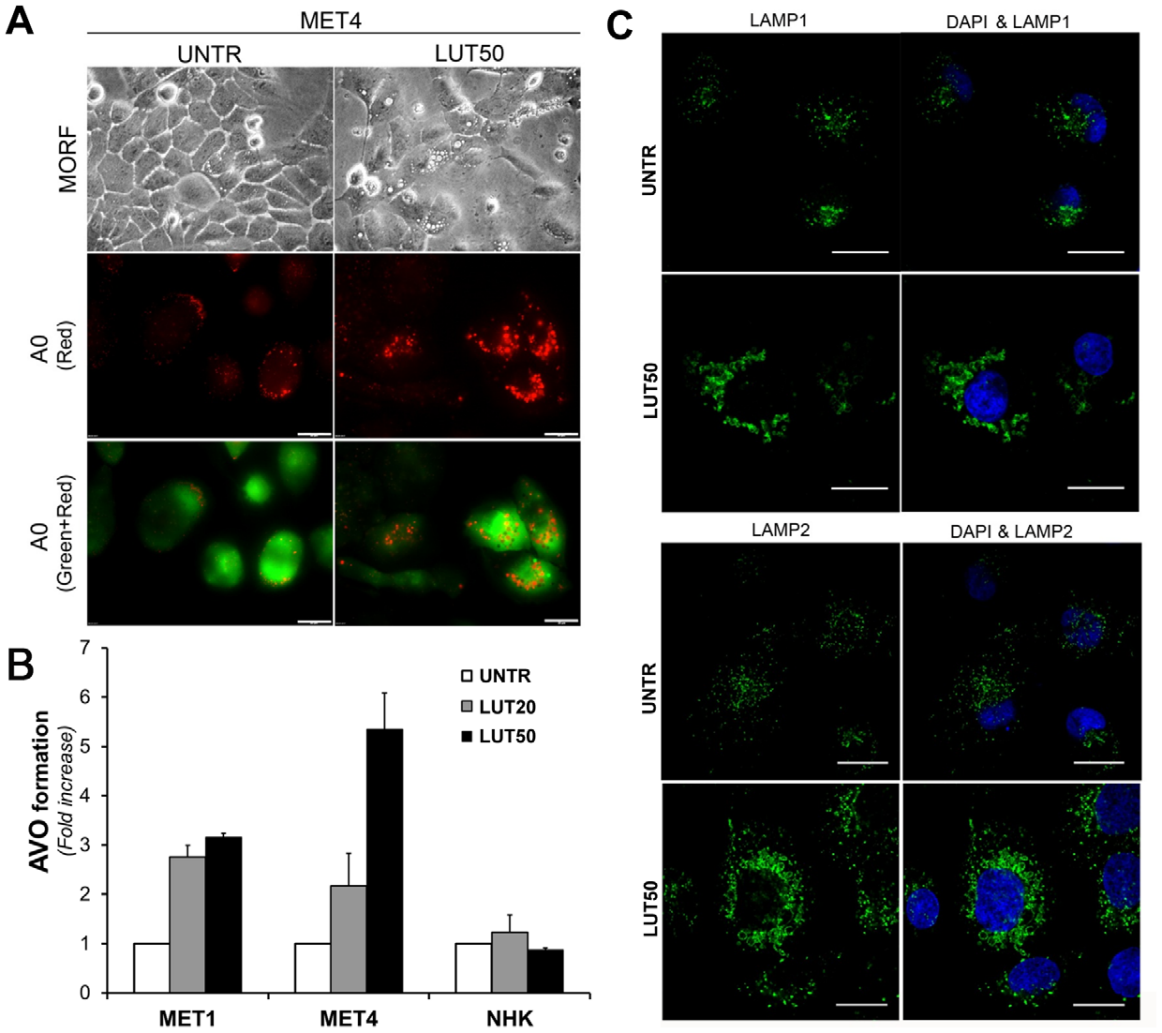
Expanded lysosomal compartment following LUT-treatment in metastatic SCC cells. **A.** *First row*: Bright field microscopic pictures of MET4 treated or not with LUT (50 μM) for 24 hours. *Second and third row*: Fluorescent microscopic images of AO stained MET4 cells treated with LUT (50 µM) or left untreated. Red  =  AVO; Green: non-acidic cell compartment (scale bar  = 20 µm). **B.** Representive flow cytometric quantification of AVO formation (increase in red fluorescence in MET1, MET4 and NHKs following treatment with indicated concentrations of LUT **C.** Confocal images of LAMP-1 and LAMP-2 stained *(green)* MET4 cells treated with LUT (50 µM) for 16 h. DAPI *(blue)* was used for nuclear staining.

Besides the typical morphological changes accompanying the apoptotic phenotype induced by LUT, we also observed in the cytoplasm of the MET4 cells the formation of vacuoles *(* *Figure* *3A* *-first row)*. Staining the LUT-treated MET4 cells with acridine orange (AO), identified those vesicles as acidic vesicular organelles (AVO) *(* *Figure* *3A* *, AVO*'*s in red)*, suggesting an increase of the lysosomal compartment. Quantification of AVO's by flow cytometric analysis of LUT-treated MET1, MET4 and NHK-cells showed a dose-dependent increase of AVO's which was more pronounced in MET4 cells. In contrast, we could not detect an increase of AVO's in NHKs *(* *Figure* *3B* *)*.

Lysosomes are crucially important for catabolic processes such as autophagy, which is a survival mechanism involved in the breakdown and recycling of damaged or potentially dangerous proteins and organelles in response to stress [22], [28]. Moreover, it was recently discovered that an increase in the number of perinuclear lysosomes makes cells more prone for autophagic flux, as fusion between autophagosomes and lysosomes becomes more likely [29], [30]. To visualize the lysosomal compartment in LUT-treated cells, we stained lysosomes using Lysosome Associated Membrane Protein (LAMP)-1 and LAMP-2 directed antibody and used DAPI for nuclear staining. These stainings revealed an increase in number and in size of lysosomes around the nucleus of MET4 cells following LUT-treatment *(* *Figure* *3C* *)*.

Hence, the observed massive increase of the lysosomal compartment in MET4 as a result of LUT treatment, suggests the stimulation of a catabolic process dependent on lysosomal degradation.

### Autophagy is involved in the response of SCC cells to LUT

**Figure 4:**
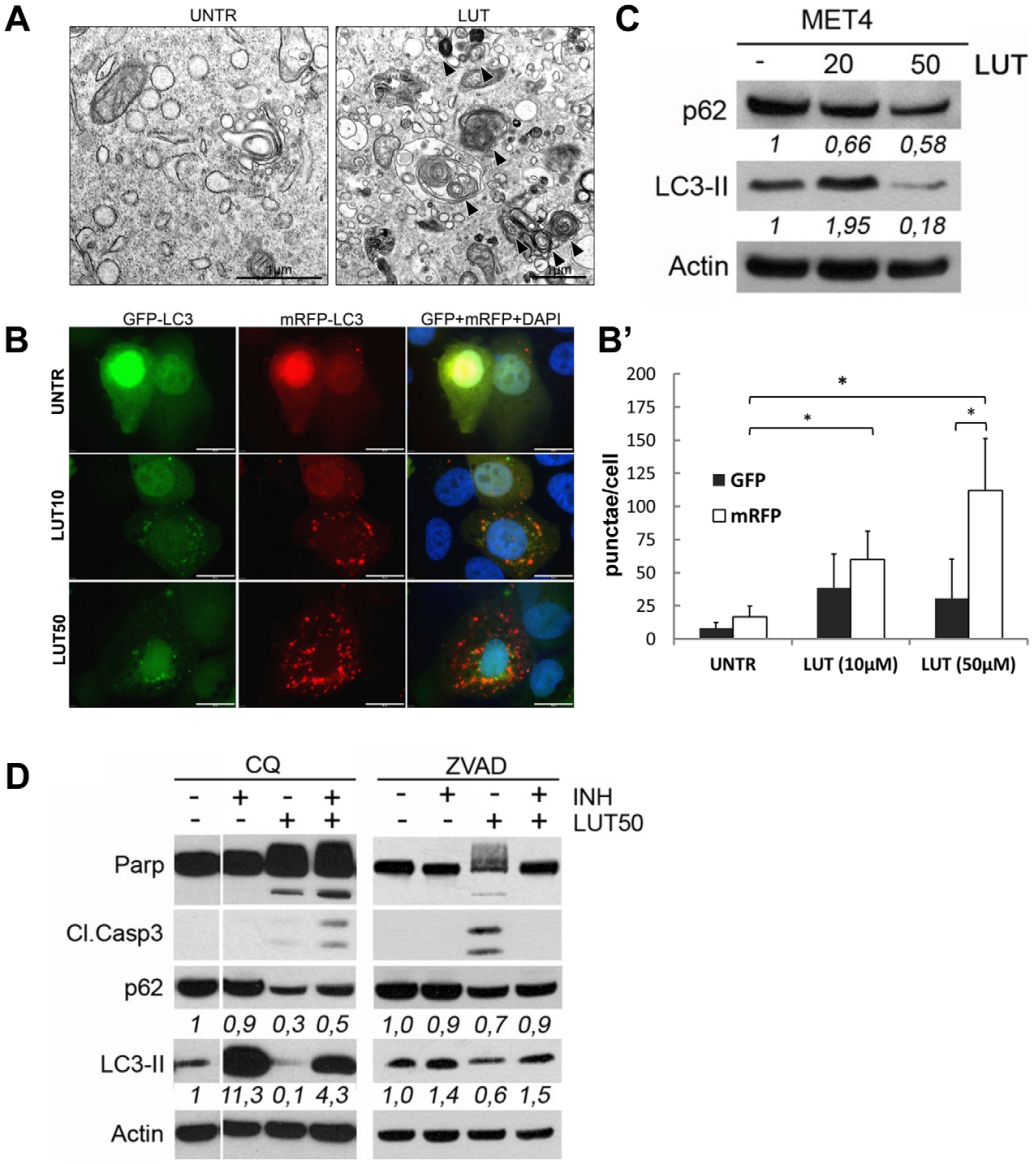
Involvement of autophagy in the response of SCC cells on LUT. **A.** Electron micrographs showing the ultrastructure of MET4-cells treated with LUT (20 µM) or not. Arrows indicate autophagosomes (scale bar  = 1 µm). **B.** *(left panel)* Fluorescent microscopy analysis of MET4 cells transiently overexpressing mRFP-GFP-LC3, treated or not with LUT (10 or 50 µM) for 24 hours (scale bar  = 20 µm). (right panel) Quantification of red and green fluorescent punctae of at least 10 cells per condition is shown. **C.** Protein levels of p62 and LC3-II upon LUT treatment in MET4 cells. One representative blot of at least three independent experiments is shown. Values are the ratio's p62 and LC3-II to actin level of densitometric analysis. **D.** Cells were pre-treated (1 hour) with an autophagy inhibitor (INH), CQ (50 µM) or apoptosis inhibitor (INH), ZVAD-fmk (50 µM), before LUT treatment (50 µM). Lysates were made after 24 hours and analysed by western blot. A representative blot of at least three independent experiments is shown. Numbers are the ratios of the densitometric analysis for p62/actin and LC3II/actin.

Reduced AKT/mTOR signaling paralleled by an increase in lysosomal activity points towards the activation of a specific catabolic process, called autophagy, which is known to be a survival mechanism frequently utilized by cancer cells to adapt to metabolic and cellular stress [31], [32]. An important hallmark of autophagy is the formation of double membrane vacuoles, called autophagosomes. Ultrastructural analysis of LUT-treated MET4 cells using transmission electron microscopy (TEM) showed increased presence of autophagosomes filled with cargo *(* *Figure* *4A* *)*.

An increase in autophagosome accumulation may result either from increased autophagosome formation or from the blockage of autophagic degradation after fusion of the autophagosome with a lysosome (autolysosome) [33]. The tandem-tagged LC3 construct (tfLC3), mRFP-GFP-LC3, is used to distinguish early and late autophagosomes. The GFP-tag will be quenched quickly in the acidic environment of the autolysosome, leaving only the mRFP-tag detectable [34]. *Figure* *4B* clearly shows the accumulation of mRFP punctae in the absence of green fluorescence in LUT-treated MET4 cells (Figure 4B), indicating an increased autolysosome formation and enhanced autophagic flux.

Autophagic flux can also be evaluated by decreased p62 protein levels, since p62 acts as an autophagosomal cargo receptor for ubiquitinated proteins, which is degraded in the autolysosome [35]. Indeed, addition of LUT to MET4 cells decreased p62 levels, albeit only at higher LUT concentrations, suggesting that LUT increases the autophagic flux *(* *Figure* *4C* *)*. Levels of LC3-II, which is incorporated in the outer and inner membrane of the autophagosomes [36], decreased as well upon treatment with 50 µM of LUT which is possibly due to the massive formation of autolysosomes and the simultaneous induction of apoptosis. In agreement, blocking lysosomal degradation using chloroquine (CQ) rescued LC3-II and p62 breakdown. Interestingly, caspase inhibition by ZVAD-fmk also increased the level of LC3-II and p62, suggesting that caspase signaling may modulate the autophagic process *(* *Figure* *4D* *)*.

In conclusion, our data indicate that LUT induced autophagy in the metastatic MET4 cells.

### Inhibition of autophagic flux sensitizes MET4 cells to LUT-induced cell death

**Figure 5:**
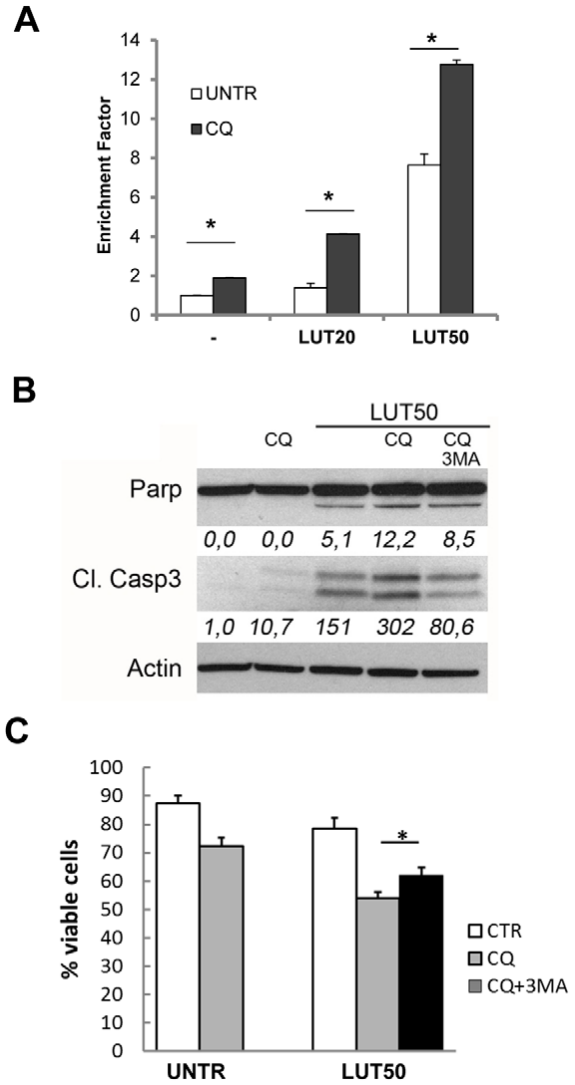
Inhibition of late phase autophagy increases LUT induced cell death in metastatic SCC. **A.** Cells were pre-treated with CQ (0 or 50 µM) and after 1 hour LUT (0, 20 or 50 µM) was added for 24 hours. The amount of apoptotic DNA was determined by the Cell death detection ELISA. Enrichment factor relative to untreated controls (UNTR/-) was calculated. Experiment was performed twice in duplicate. **B.** Western blot of cell lysates of MET4 cells pre-treated for 1 hour with CQ (50 µM) and/or 3-MA (10 mM) before the addition of LUT (50 µM) for 24 hours. Densitometric analysis of PARP% (cleaved Parp/(total Parp + cleaved Parp) and of cleaved caspase 3 relative to actin level is shown. Representative blot of three independent experiments is shown. **C.** Trypan blue exclusion assay of MET4 cells treated as indicated and described in **B.** (Experiment performed twice, n = 4).

Since autophagy, and more specifically formation of autolysosomes, was increased in MET4 cells upon treatment with LUT, we hypothesized that blockage of late phase autophagy might make the MET4 cells more susceptible to LUT-induced apoptosis. We therefore used the late phase autophagy inhibitor chloroquine (CQ) (which prevents the autophagosomal degradation) [37] in combination with LUT and evaluated apoptosis induction. Treatment of MET4 cells with CQ and LUT, resulted in a significant increased induction of apoptosis *(* *Figure* *5A* *)* and in enhanced caspase-3 and Parp cleavage *(* *Figure* *5B* *)* compared to treatment with LUT alone. Inhibition of autophagy at an early stage using 3-methyl adenine (3-MA) simultaneously with CQ and LUT rescued partially the apoptosis-inducing effect of CQ *(* *Figure* *5B*–*5C* *)*.

These results suggest that autophagy serves as a cytoprotective mechanism against LUT-mediated cellular damage in the advanced metastatic MET4 SCC cells.

## Discussion

In this study, we showed for the first time that the promising anticarcinogenic flavonoid Luteolin (LUT) decreased AKT/mTOR signaling and in parallel induced caspase-dependent cell death in both primary and metastatic cutaneous squamous cell carcinoma (SCC) cells. LUT-induced apoptosis was selective for cancer cells as normal keratinocytes (NHKs) were resistant to treatment with LUT. Additionally, we found that primary SCC cells showed a higher sensitivity to LUT than the metastatic MET4 cells. The increased resistance of metastatic cells could be explained by the contribution of the autophagic process as a survival mechanism. Inhibition of autophagy using chloroquine (CQ) together with LUT treatment in MET4 cells decreased viability significantly more than addition of LUT alone.

Cutaneous SCC is a very common cancer in the Caucasian population [38] and although most SCCs can be cured, a subset of patients, like organ transplant recipients (OTR) develop advanced SCC with a high risk of metastasis [39]. Metastatic SCC has a poor prognosis because of its resistance against the classical chemotherapeutic agents [40], [41]. Hence, the identification and validation of new therapeutic approaches that circumvent chemoresistance and thus improve outcome of patients with more advanced stages of SCC is of greatest importance.

One approach would be the use of flavonoids, naturally occurring compounds of which LUT is an example. LUT has been shown to exhibit anti-cancer activity *in vitro* and *in vivo* against several forms of cancers. *In vitro* studies demonstrate relevant effects on cellular proliferation, apoptosis, invasion, angiogenesis and metastasis. Mice studies support an anticancer effect of LUT *in vivo*. LUT enhances TNF-related apoptosis inducing ligand's anticancer activity in a lung cancer xenograft mouse model [42]. After topical application prior to TPA treatment, LUT inhibits tumor promotion in DMBA-initiated mouse skin [43]. LUT also inhibits lung metastasis of prostate cancer cells, implanted into nude mice [27], [44].

Although the cytotoxic effects of LUT have been reported in several studies, the underlying mechanism of LUT's cytotoxicity is complex, not yet completely understood, seems to differ between cancer cell types and appears to be selective for cancerous cells [1], [27]. We have shown previously that LUT was able to protect normal but not malignant human keratinocytes against UVB-induced cell death [9]. In the current study, we used cancer cells derived of a primary, invasive SCC (MET1) and of its lymph node metastasis (MET4) from an immune suppressed patient to study the effects of LUT. We could show that treatment with a broad concentration range of LUT (50–100 µM) for 24 hours induced cell death in, primary and metastatic cutaneous SCC cell lines. Normal keratinocytes on the other hand remained viable up to at least 100 µM LUT, suggesting a selective toxicity for malignant keratinocytes, which is an advantage for LUT's possible use as a chemotherapeutic. Nevertheless 50 μM LUT remains a high dose and may be toxic when given systemically to reach these concentrations locally in the skin. Indeed more preclinical work, including animal studies, investigating safety and efficacy of systemic administration of LUT, is needed before Luteolin can be tested in clinical trial as a SCC-therapeutic. Since an in vivo study has demonstrated that LUT is not only absorbed at the skin surface but even penetrates into the deeper skin layers [45], the efficacy of local application of LUT, or intralesional injection, could be tested in animal studies. In this way side effects due to systemic administration could be avoided.

Earlier work in this unique model of skin cancer progression revealed that AKT signaling gained importance in the subsequent SCC cancer progression stages [24]. Since the PI3K/AKT pathway is essential in the determination and regulation of cell fate and is considered to be able to induce oncogenic transformation and chemoresistance, AKT represents an interesting molecular therapeutic target [13], [16]. Indeed, we showed earlier that specific inhibition of AKT sensitized MET1 and to lesser extent MET4 cells to apoptosis induced by cisplatin, a chemotherapeutic agent currently used in the treatment of metastatic SCC. Therefore, we tested whether LUT was able to influence AKT-signaling by checking AKT levels and activation status. Here we show that LUT treatment rapidly and drastically inhibited AKT phosphorylation and signaling in both MET1 and MET4 cells. Moreover, further reduction of AKT signaling using a specific AKT inhibitor or ectopic expression of a constitutive active AKT mutant demonstrated that these SCC cells depend highly on AKT signaling for their survival. Although in both MET1 and MET4 cells the combined treatment with AI and LUT resulted in an additional induction of apoptosis the MET1 cells appeared much more sensitive to AI and/or LUT induced cytotoxicity. These results indicated that the more advanced MET4 cells probably activate additional survival mechanisms, such as autophagy.

In agreement with this hypothesis, we found that addition of LUT (≥20 µM) to MET4 cells caused the appearance of an intense cytoplasmic vacuolization, which was detectable using bright field microscopy. In line with the increase of lysosomes in the perinuclear region and with the inhibition of AKT/mTOR signaling, we detected increased number of autophagosomes using ultrastructural TEM analysis in LUT-treated MET4 cells. Interestingly, recently it was suggested that the availability of lysosomes in the perinuclear region is a determining factor for the fusion between autophagosomes and lysosomes, and thus for the fulfillment of the autophagy process [30]. In agreement, we found that LUT dose dependently increased autophagic flux in MET4 cells.

Autophagy is generally used by (cancer) cells as a survival mechanism in an attempt to cope with extrinsic or intrinsic stresses by recycling cellular components [31]. Therefore, a higher basal level of autophagy is often seen in malignant cells due to their high metabolic state [46]. However, bulk destruction of cellular content by autophagy [21], [32] or even leakage of lysosomal cathepsins into to the cytoplasm [28], [47], may lead to autophagic cell death or apoptosis. Moreover, the regulation of these processes is intimately connected and partly regulated by the same signaling pathways, including PI3K/AKT/mTOR. Our results show that AKT seemed to play a dual role in our study; while AKT inhibition was linked to apoptosis induction in MET1 cells; it was associated with the induction of autophagy in MET4. The underlying mechanisms of this dual role of AKT in the progression of cutaneous SCC was beyond the scope of this study and needs to be more systematically addressed in further research.

While numerous studies have evaluated the cell death inducing effect of several flavonoids in cancer cells [48], [49], the effects of flavonoids on autophagy are poorly documented. However, massive acidic vacuolization was observed upon *in vitro* treatment of cells with prenylflavonoids [50], [51], Quercetin [52] and Curcimin [53]. Induction of autophagy by flavonoids or flavonoid-containing multiherbal preparation was seen to induce apoptotic [54], [55] and non-apoptotic, caspase independent cell-death [50], [51]. Moreover, blocking of autophagosome maturation by flavonoids was seen to result in apoptosis recently [56]. Considering these opposite effects, specified research is necessary to unravel the diverse effects of flavonoids on autophagy and apoptosis. We report for the first time the induction of autophagy in a metastatic SCC cell line (MET4) following treatment with the flavonoid LUT.

Furthermore, we assessed the contribution of LUT-dependent autophagy induction to the increased survival of MET4 cells using the lysosomotropic agent chloroquine, which prevents lysosomal degradation. The marked increase of cell death detected upon CQ co-treatment, suggested that addition of a late phase autophagy inhibitor to LUT reduced chemoresistance of metastatic SCC. Notably, addition of 3-MA, an early stage autophagy inhibitor, diminished the improved apoptosis induction by CQ in LUT treated cells, suggesting that accumulation of lysosomes is necessary for the cell death inducing effect of LUT. A different outcome of autophagy inhibition depending on the stage of autophagy inhibition (early versus late), has been reported earlier in an Imatinib study [57]. The combination of an autophagy inhibitor with agents that induce autophagy in cancer cells as a survival response has been proposed recently as a novel cancer therapeutic strategy. For instance, combination of CQ or hydroxychloroquine (HCQ) with chemotherapy, targeted therapy or radiotherapy is currently under intensive investigation in preclinical as well as clinical trials [58], [59]. Thus, cancer appears to be a promising indication for this old drug CQ, which is widely used as an anti-malarial and anti-rheumatic drug.

In summary, different human malignant keratinocyte cell lines (SCC) treated with LUT showed increased apoptotic cell death, while normal human keratinocytes remained unaffected. In addition, the efficacy of LUT to induce apoptosis appeared to be tumor progression dependent. Whereas primary MET1 tumor cells were very sensitive to AKT-inhibition by LUT, complete inhibition of AKT signaling in metastatic MET4 cells was not sufficient for notable cell death induction. However, LUT induced massive autophagy in MET4 cells, which was paralleled by a vast increase in acidic vacuoles. We showed that simultaneous inhibition of late phase autophagy resulted in a sensitization of the resistant MET4-cells to LUT-induced cell death. Altogether, our *in vitro* data suggest that LUT may provide a therapeutic tool for the treatment of patients with advanced SCC and that resistance may be circumvented by supplementation of autophagy inhibitors, like CQ. Additional studies are warranted to further investigate the therapeutic value and clinical usefulness of LUT.
